# Concomitant glenohumeral pathologies in high-grade acromioclavicular separation (type III – V)

**DOI:** 10.1186/s12891-017-1803-y

**Published:** 2017-11-10

**Authors:** Jochen Markel, Tim Schwarting, Dominik Malcherczyk, Christian-Dominik Peterlein, Steffen Ruchholtz, Bilal Farouk El-Zayat

**Affiliations:** Center of Orthopaedics and Traumatology, University Hospital Marburg, Baldingerstrasse, 35033 Marburg, Germany

**Keywords:** Acromio-clavicular joint separation, Concomitant injuries, Shoulder arthroscopy

## Abstract

**Background:**

Acromioclavicular joint (ACJ) dislocations are common injuries of the shoulder associated with physical activity. The diagnosis of concomitant injuries proves complicated due to the prominent clinical symptoms of acute ACJ dislocation. Because of increasing use of minimally invasive surgery techniques concomitant pathologies are diagnosed more often than with previous procedures.

**Methods:**

The aim of this study was to identify the incidence of concomitant intraarticular injuries in patients with high-grade acromioclavicular separation (Rockwood type III – V) as well as to reveal potential risk constellations. The concomitant pathologies were compiled during routine arthroscopically assisted treatment in altogether 163 patients (147 male; 16 female; mean age 36.8 years) with high-grade acromioclavicular separation (Rockwood type III: *n* = 60; Rockwood type IV: n = 6; Rockwood type V: *n* = 97).

**Results:**

Acromioclavicular separation occurred less often in women than men (1:9). In patients under 35, the most common cause for ACJ dislocation was sporting activity (37.4%). Rockwood type V was observed significantly more often than the other types with 57.5% (Rockwood type III = 36.8%, Rockwood type IV 3.7%). Concomitant pathologies were diagnosed in 39.3% of the patients with that number rising to as much as 57.3% in patients above 35 years. Most common associated injuries were rotator cuff injuries (32.3%), chondral defects (30.6%) and SLAP-lesions (22.6%). Of all patients, 8.6% needed additional reconstructive surgery.

**Conclusion:**

Glenohumeral injuries are a much more common epiphenomenon during acromioclavicular separation than previously ascertained. High risk group for accompanying injuries are patients above 35 years with preexisting degenerative disease. The increasing use of minimally invasive techniques allows for an easier diagnosis and simultaneous treatment of the additional pathologies.

## Background

The Acromioclavicular joint (ACJ) is a diarthrodial joint between the distal clavicle and acromion. There is no active motion of the AC joint. Joint movement occurs when the clavicle rotates passively during alteration of scapula placement [1]. Joint stability is achieved with musculoskeletal and ligamentous stabilizers such as the deltoid and trapezoid muscle, capsula, acromioclavicular and coracoclavicular ligaments [2]. During acromioclavicular separation the ligamentous structures rupture first. With higher force the musculoskeletal structures rupture as well [3, 4]. This progression of injury is illustrated in several classification systems of ACJ dislocation (see below).

The occurrence of acromioclavicular joint dislocation highly correlates with physical activity. While injuries of the acromioclavicular joint account for only 3–12% of all shoulder injuries, the incidence rises during sporting activity to up to 40% in contact sports (e.g. Football) [4, 5]. ACJ-dislocation occurs 5 to 10 times more often in men than in women. High risk groups are young and active men [4, 6].

Several classification systems have been described for ACJ separation, starting in 1917 with Cadenat [7]. Today the most commonly used classifications are the Tossy classification of 1963 [8] and the subsequent Rockwood classification of 1984 [9] which adds the aspect of horizontal instability. Both are primarily radiological classifications. The higher the classification, the graver the injury and hence the more likely the stabilizers rupture.

Whereas Rockwood type I and II injuries can almost always be treated conservatively, Rockwood type IV to VI injuries require surgical treatment. Unfortunately there is still discord about the treatment of Rockwood type III injuries in literature. Older studies suggest a similar or even better result of the conservative treatment [10–13]. A consensus of the ISAKOS (International Society of Arthroscopy, Knee Surgery and Orthopedic Sports Medicine) recommends conservative treatment of Rockwood type III injuries and re-evaluation in 3 to 6 weeks [14]. In recent years studies show that early treatment especially in young and active patients improves the clinical outcome [15, 16]. In Germany, there is an increasing tendency to treat Rockwood type III injuries surgically. In a nationwide poll 73% of all queried hospitals stated to prefer surgical treatment to conservative treatment. The most commonly used techniques in Germany are the hook plate with 44% and the arthroscopic TightRope™ with 27% [17]. In comparison of open and minimally invasive techniques no significant difference in outcome has been established [18].

Pauly et al. and Tischer et al. (both 2009) showed that 15.0% to 18.2% of all patients with acromioclavicular separation had suffered concomitant intraarticular injuries (altogether 20 of 117) [19, 20]. Typical concomitant injuries were rotator cuff injuries, SLAP-lesions (Superior Labral tear, Anterior to Posterior) or fractures.

Aim of this study was to reassess this data in a bigger collective and establish the incidence of concomitant lesions in acromioclavicular separation and preeminent risk constellations.

## Methods

Between the years 2009 and 2015 163 patients (147 male; 16 female; mean age 36.8 years) suffering from high-grade acromioclavicular separation (Rockwood type III: *n* = 60; Rockwood type IV: n = 6; Rockwood type V: *n* = 97) underwent arthroscopically assisted treatment during which the pathologies were compiled. The obtained data was analyzed descriptively, statistically and the causality of the concomitant pathologies were attributed to three factors (acute, intermediate and degenerative causes). Acute pathology was defined where the trauma was the sole cause for the diagnosed pathology (Intraoperative hemarthrosis and/or reddish blood tinged pathologies), intermediate pathology had a mixed etiology, and degenerative pathology showed mostly preexisting defects which were only slightly aggravated by the injury. Several subgroup analyses concerning the severity of the AC-separation, sex and age of the patient were conducted.

The data was analyzed with SPSS for Windows, Version 22 (SPSS, Chicago, IL, USA). Probability distribution was determined with the Kolmogorov-Smirnov test. Statistical significance was calculated with the Chi-Square test and in the case of small case numbers, Fisher’s exact test was used.

## Results

ACJ dislocation was predominantly diagnosed in men (9:1; *p* < 0,001).

**Fig. 1:**
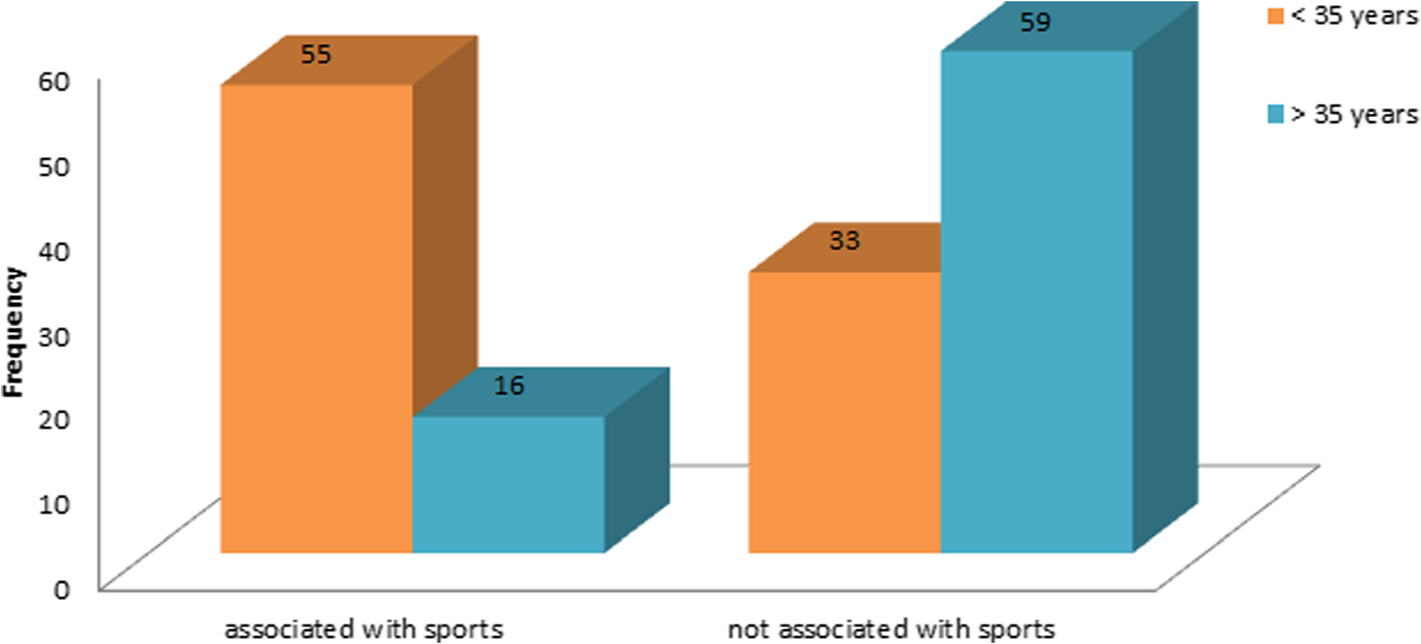
Association with sporting activity

Cause of ACJ-dislocation was in 37.4% sporting activity for all patients. Young patients below 35 years suffered an acromioclavicular separation significantly more often during sports (54.5%, *n* = 48) than patients above 35 (*p* < 0,001; see Fig. 1).

**Fig. 2:**
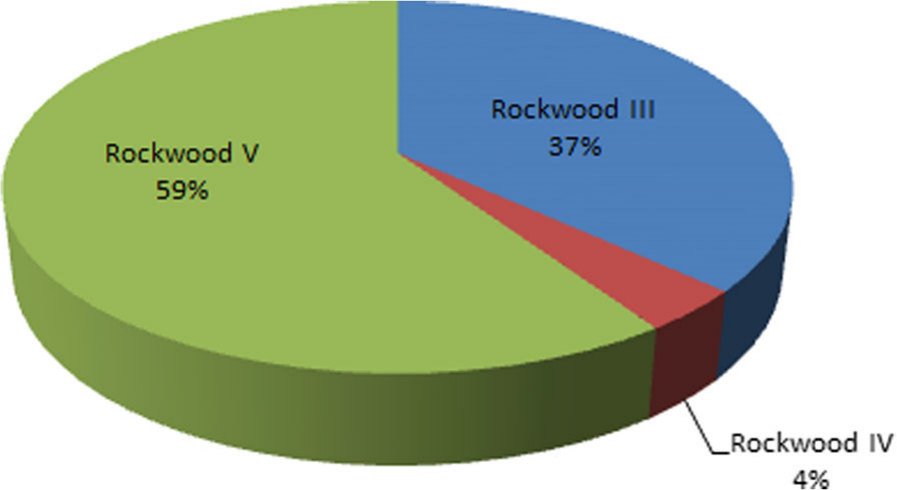
Rockwood grade

3.7% (*n* = 6) of all patients suffered a Rockwood type IV injury, 36.8% (*n* = 60) Rockwood type III while Rockwood type V injuries were observed significantly more often than the other grades (*p* = 0,015) with 57.5% (*n* = 97; see Fig. 2).

**Fig. 3:**
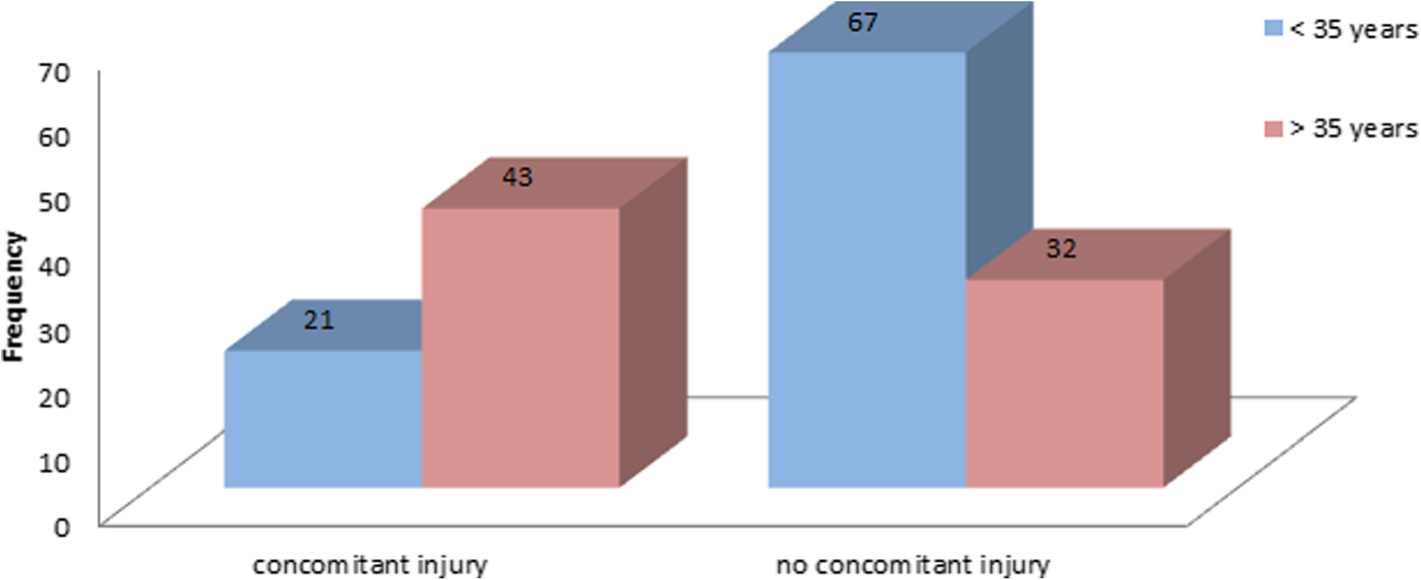
Concomitant pathologies

Concomitant pathologies were found in 39.3% (*n* = 64) of the patients with that number rising to as much as 57.3% (*n* = 43) and significantly higher (p < 0,001) in the age group above 35 years (see Fig. 3).

An average of 1.9 injuries per patient with a total of 124 accompanying pathologies occurred. In the following the absolute total of pathologies will be counted, not considering the fact that in many patients several concomitant injuries at once could be found. Typical constellations were chondral defects on the humerus as well as the glenoid and several rotator cuff injuries at once. Of these 64 patients, 21.9% (*n* = 14) needed additional reconstructive surgery (e.G. *rotator* cuff and SLAP repair).

The incidence of rotator cuff injuries accounted for 32.3% (*n* = 40) of all concomitant injuries.

**Fig. 4:**
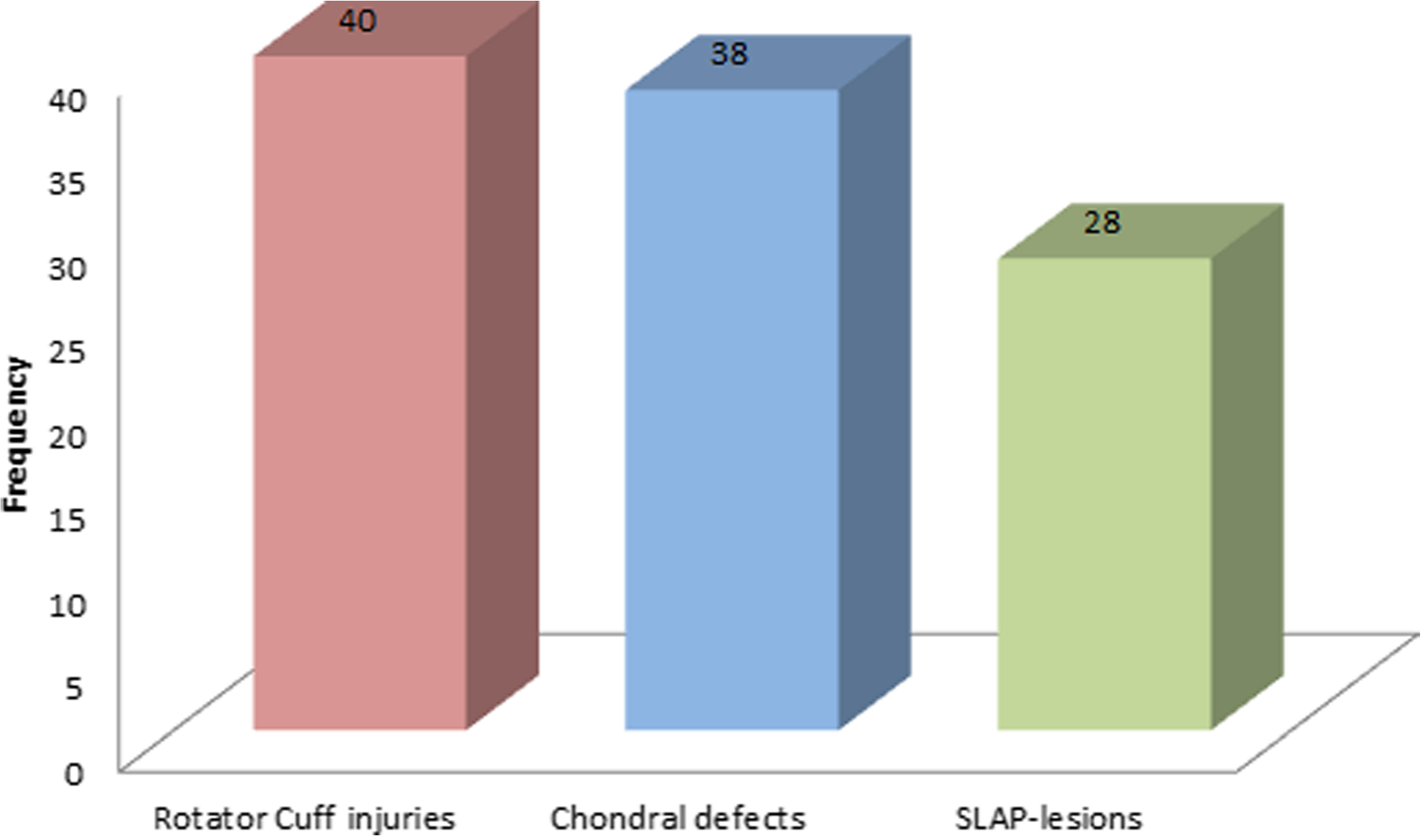
Types of pathologies

Chondral defects were diagnosed in 30.6% (*n* = 38) and SLAP-lesions in 22.6% (*n* = 28) of all injuries (see Fig. 4). Accompanying pathologies were attributed to preexisting degeneration in 70.0% (*n* = 42) of the cases.

Subgroup analysis showed no difference concerning etiology, kind and frequency of concomitant injuries during AC-separation between the sexes and between the different Rockwood types.

Specific concomitant pathologies in the subgroup of patients above 35 years were SLAP-lesions (*p* < 0,001), lesions of the M. subscapularis (*p* < 0,001), injuries of the long biceps tendon (*p* = 0,002) and glenoidal chondral defects (p = 0,029). All pathologies in the age group above 35 years were significantly more likely to have been degenerative (*p* < 0,001).

## Discussion

To date there are only two publications discussing concomitant injuries in ACJ separations, with small collectives. In the present study a collective of 163 patients with ACJ separations is presented.

As in recent literature [4, 6], men in the present collective were much more likely than women to having suffered acromioclavicular separation (p < 0,001).

Etiology of ACJ-dislocation was sporting activity in 37.4% for both sexes and all age groups. In the younger age group acromioclavicular separation occurred significantly more often (p < 0,001) during sporting activity (54.5%). This is also consistent with current data [4, 5]. In the older age group ACJ separation did not necessarily occur during sporting activity and no predominant trauma mechanism could be found. With higher age the rate of preexisting degenerative defects is higher as well. Therefore a rising incidence of ACJ separation even during minor trauma is plausible.

In our study group Rockwood type V injuries were most common with 57.5%. Recent studies have shown mostly Rockwood type III injuries as the most common type of acromioclavicular separation [10]. Since only surgically treated patients were included into the study group the real total of Rockwood type III patients will most likely be higher. There is no definite conclusion about accompanying injuries in all Rockwood III patients (regardless if conservatively or surgically treated). Additional studies concerning concomitant pathologies in conservatively treated Rockwood III cases should be performed (e.g. MRI studies).

Most interestingly there was no difference of frequency or etiology of concomitant pathologies between the different Rockwood types. The patho-mechanism of the ACJ separation would suggest that with rising force during impact it is more likely for intraarticular lesions to occur. In our study group this was not the case.

To date there are only two studies which compiled the frequency of intraarticular lesions associated with ACJ separation. These studies by Tischer et al. (2009) [20] and Pauly et al. (2009) [19] show a combined 17.1% (*n* = 20/117) of concomitant injuries. In this study group, concomitant pathologies were found in 39.3% (*n* = 64) of all patients with that number rising to as much as 57.3% (*n* = 43) and thus significantly higher (*p* < 0,001) in patients above 35 years. A contributing factor may be that in the studies mentioned above only the most relevant accompanying injuries were collected (e.g. no chondral defects). If omitting chondral defects for comparison, the rate of accompanying pathologies would still be 29.4% in the present study collective. Biggest discrepancy was found in prevalence of rotator cuff lesions which were diagnosed more often in this collective, whereas SLAP-lesions occurred in comparable frequency. Possibly, the slightly higher mean age in this group is a contributing factor to the higher rate of rotator cuff lesions.

8.6% of all patients needed reconstructive surgery (e.G. *rotator* ruff- and/or SLAP-repair) in addition to the treatment of the ACJ dislocation. If surgery had not been performed arthroscopically, it is likely that at least in some cases diagnosis and treatment would have been delayed/not performed.

Accompanying pathologies were sorted into the above mentioned three groups. Most commonly preexisting degeneration was found in the age group above 35 years (70.0%). As expected, no dominant etiology of the pathology was detected in patients below 35 years.

The subgroups for sex and the different Rockwood types showed no difference concerning etiology, kind and frequency of concomitant pathologies.

## Conclusion

The present study shows that the incidence of concomitant injuries in acromioclavicular separation has been underestimated. In almost 40% of all patients with ACJ-dislocation a concomitant injury could be diagnosed during the preceding diagnostic arthroscopy. In the age group above 35 years the incidence of an accompanying injury rises to 57.3%. Most commonly diagnosed pathologies were rotator cuff injuries, SLAP-lesions and chondral defects. 70% of all concomitant injuries could be attributed to a mostly degenerative etiology. Preeminent risk group to having suffered an accompanying injury are patients above 35 with degenerative defects. 21.9% of all patients with concomitant pathology needed additional reconstructive surgery.

In patients with ACJ separation diagnostic shoulder arthroscopy should be integrated as standard procedure for detection and treatment of concomitant pathologies.

## Abbreviations

AC: Acromioclavicular
ACJ: Acromioclavicular Joint
ISAKOS: International Society of Arthroscopy, Knee Surgery and Orthopedic Sports Medicine
M: Musculus, muscle
MRI: Magnetic Resonance Imaging
SLAP: Superior Labral tear, Anterior to Posterior
